# Quantifying photosynthetic restrictions

**DOI:** 10.1007/s11120-024-01129-y

**Published:** 2025-02-18

**Authors:** Chandra Bellasio

**Affiliations:** 1 Laboratory of Theoretical and Applied Crop Ecophysiology, Department of Chemistry, Biology and Biotechnology, Università Degli Studi Di Perugia, 06122 Perugia, Italy; 2 School of Biology and Environmental Science, University College Dublin, Belfield, Dublin, Ireland; 3 Biology of Plants Under Mediterranean Conditions, Department of Biology, University of the Balearic Islands, Illes Balears, 07122 Palma, Spain; 4 Research School of Biology, The Australian National University, Acton, ACT 2601 Australia

**Keywords:** Modelling, Partitioning, COntribution, Limitation, Control, Sensitivity, Stomatal, Non-stomatal, Mesophyll, Biochemical, Non-diffusional

## Abstract

Quantifying the effect of factors controlling CO_2_ assimilation is crucial for understanding plant functions and developing strategies to improve productivity. Methods exist in numerous variants and produce various indicators, such as limitations, contributions, and sensitivity, often causing confusion. Simplifications and common mistakes lead to overrating the importance of diffusion—whether across stomata or the mesophyll. This work develops a consistent set of definitions that integrates all previous methods, offering a generalised framework for quantifying restrictions. Ten worked examples are provided in a free downloadable spreadsheet, demonstrating the simplicity and applicability to a wide range of questions.

**Supplementary Information:**

The online version contains supplementary material available at 10.1007/s11120-024-01129-y.

## Introduction

Measurements of photosynthetic gas exchange routinely quantify leaf CO_2_ uptake (assimilation,* A*) and water vapour release (transpiration, *E*), under step-wise or continuous changes in external CO_2_ concentration or light intensity (Busch et al. 2024). Raw data is first processed by the software embedded in the analyser, to derive stomatal conductance to CO_2_ (*g*_S_), together with the CO_2_ mole fraction in the substomatal cavity (*C*_i_) using mass-balance and diffusion theory (von Caemmerer and Farquhar 1981). The resulting ordered pairs (*C*_i_, *A*) can be further processed.

One goal is to estimate parameters that capture physical or biochemical characteristics of leaves. This is typically achieved through curve fitting procedures whereby mathematical models of photosynthesis are adjusted to match data (Bellasio et al. 2016a, b). A variety of models exist that are typically classified as mechanistic or empirical. Mechanistic models have expressions that describe biochemical processes known to occur within leaves, previously validated by direct measurements of physical or biochemical properties. They are modular so that they can be modified to include more processes, and the description of processes can be refined to any desired level of detail, allowing them to accommodate a virtually unlimited range of real or hypothetical scenarios. Empirical models have expressions that describe the relationship between data patterns. They are generally simpler, making them typically more accurate for describing data and valid for all photosynthetic types, but they may become unreliable when used in environmental conditions differing from those under which they were calibrated. The distinction is not clear-cut, because mechanistic models still rely on data, while empirical models describe patterns that ultimately depend on those same underlying processes.

Another goal is to gain insights into how the state of the assimilation process is perturbed or restrained. Methods based on the theoretical basis laid by Gaastra (1959) have been developed over the years with different assumptions, conventions, and resulting issues. Early studies made the erroneous assumption that CO_2_ mole fraction at the mesophyll carboxylating sites (*C*_M_) is zero. In the 1970s, biochemical CO_2_ uptake was treated as a linear function akin to Ohm’s Law of electrical resistance (Jarvis 1971; Jones 1973; Jones and Slatyer 1972; Prioul and Chartier 1977). This ‘linear resistance analysis is in most cases invalid’ (Farquhar and Sharkey 1982) because photosynthesis typically responds non-linearly with a saturating response to CO_2_. Besides, rather than conveying information about the process, resistance is confounded with a physical characteristic that is difficult to relate to photosynthetic biochemistry, making it difficult to extract the desired process information. Linear resistances are therefore undesirable and will not be addressed further. Alternatively, the concept of restriction was related to the extent to which various input variables exert control over the process of assimilation, akin to sensitivity analysis. This has the benefit that, once model parameterization is defined, outputs are unequivocal (Jones 1973). The drawback is that control coefficients are relatively complex to derive, often difficult relate to actual physiological phenomena, and are applicable only to infinitesimal change. Extending sensitivity analysis to finite intervals between two well-identifiable conditions of interest has the benefit of dealing with an intuitive metric—the change in assimilation—but requires approximating the response of assimilation to CO_2_ mole fraction at the mesophyll carboxylating sites (*A*/*C*_M_ curve) with a straight line. This practice introduces error (Deans et al. 2019) and is fundamentally mistaken (Buckley and Diaz-Espejo 2015), but has been widely used both for C_3_ (Grassi and Magnani 2005) and C_4_ plants (Cano et al. 2019). When the slope of that line is incorrectly derived by curve fitting under low CO_2_ concentration where it reaches its maximum value, as in Tomás et al. (2013), error is further amplified. This leads to an overestimation of the importance of stomatal or mesophyll diffusion in restricting assimilation. The solution proposed by Prioul et al. (1984) was to approximate the *A*/*C*_M_ curve with many small linear steps; however, the need to derive assimilation graphically and to process data manually, meant that most studies calculated assimilation in only three points. This raised another issue that the order in which these points are evaluated influences the result of the analysis (path dependency). The lack of an objective criterion for their selection often rendered the choice of methods ‘to some extent arbitrary’ (Jones 1985).

Recent key advancements in restriction analysis have been switching from a graphical to a computerised method where drawn curves were replaced by fitted empirical models (Bellasio et al. 2016b). The innovation of Bellasio et al. (2016b) was later utilized to compare C_4_ and C_3_ plants but remained limited in that it still used the classical three-point calculation, and was only applied to stomatal and non-stomatal limitations (Bellasio et al. 2018). Buckley and Diaz-Espejo (2015) developed over the intuition of Prioul et al. (1984) by increasing the number of steps to any large number of infinitesimal transitions. Their method had the main benefits of being unambiguous, avoiding linearization of assimilatory responses over finite intervals, and implicitly containing sensitivity, thereby reconciling contribution with control analysis. However, it only applied to contribution analysis in C_3_ plants, leaving the need for a method that is universally applicable, straightforward to implement and unambiguous.

This study develops a unified theory and terminology that can be used for control, limitation and contribution analyses in all plants, and shows the implementation of common procedures in spreadsheets freely available for download, demonstrating the framework’s simplicity and applicability to a wide range of questions.

### Theory: integrating control, limitation, and contribution analyses

Previous analyses of photosynthetic restrictions have followed three approaches.

The first termed here ‘control analysis’ quantifies the extent to which various input variables (such as *C*_i_) exert control over the process of assimilation, to derive control coefficients that are, mathematically, the absolute sensitivity and relative sensitivity (elasticity); for review see Jones (1985).

**Fig. 1:**
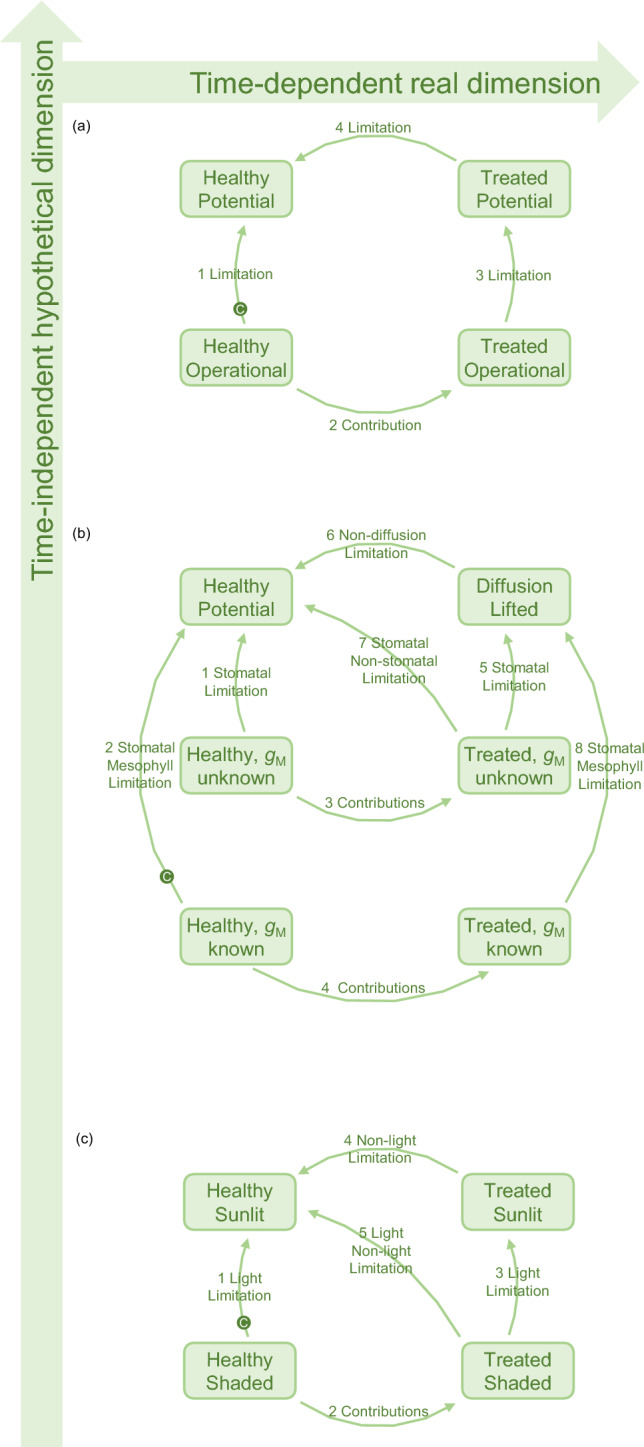
Generalised framework of restriction analysis. Restrictions may be evaluated along a real dimension (quantities change in time) and a hypothetical dimension (restrictions are lifted instantaneously). The analysis of limitations quantifies the restriction independently in each plant, compared their maximum potential. The analysis of contribution looks at the transition between two real conditions. The analysis of control (sensitivity and elasticity) can be done at any intermediate points of the transitions (exemplified by a ‘C’ in a green circle that may be swinging over all arrows). **a** General framework; **b** diffusional and non-diffusional restrictions; **c** light and non-light restrictions

The second, called ‘limitation analysis,’ quantifies the reduction in assimilation that a leaf photosynthesising in a certain state (for instance, a healthy plant photosynthesising under ordinary operational conditions) could overcome if limitations were sequentially but instantaneously lifted until photosynthesis reaches its potential maximum limit under idealised conditions (number 1 in Fig. 1a).

The third, called ‘contribution analysis,’ compares a healthy plant with the same or a similar plant after the imposition of a treatment over time. Consider, for instance, a set of plants subjected to a generic treatment and left to photosynthesise under the same ordinary operational conditions as the healthy plants. Contribution analysis assigns the total change in assimilation to the various quantities responsible for causing the change thus partitioning the change into homogeneous contributions driven by the processes affected by the treatment (number 2 in Fig. 1a).

I will now develop a generalised framework whose strength lies in its ability to combine limitation and contribution analyses with control analyses, in any nuance. Analyses can be performed independently, as in the three cases above, or combined. For instance, in a time-course experiment where a plant undergoes progressive treatment and is sampled at subsequent time points, limitation analysis can be performed by lifting limitations instantaneously at each time point (number 1 and 3 in Fig. 1a), while contribution analysis can be used to simulate the effect of the treatment by comparing these subsequent time points (number 2 in Fig. 1a). Control analysis can be overlaid at any given intermediate point along transitions (a ‘C’ in Fig. 1a) to determine the extent to which an infinitesimal change in the transition is controlled by environmental drivers and the underpinning photosynthetic characteristics.

To formalize this framework, it is necessary to assume that photosynthesis can be described mathematically. Let the function *f* be a set of expressions describing the dependence of any generic output variable Ω on a set of inputs, which are termed *a* (a generic input) and ***b*** (the set of all other inputs):

1 $$ {\Omega }_{ } = f\left( {a,{\varvec{b}}} \right) $$

**Fig. 2:**
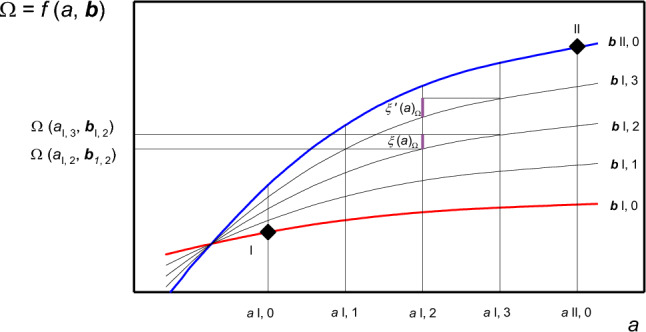
Schematic of the generalised framework of definitions. A generic output variable Ω is described through a mathematical model as a function of the variable *a* (in abscissa) and a set of other inputs ***b***. The conditions in which *a* and **b** are known are called ‘states’, here represented by two black diamonds. The function $\Omega =f\left(a, {\varvec{b}}\right)$ parameterised with the known vector ***b***, for state I is shown in red, and for state II is shown in blue. The transition between the two known states is divided in four equal intervals (*K*=0, 1, 2, 3), which are bordered by thin black lines. The value of the inputs *a* and ***b*** is assumed to vary linearly in the intervals. The marginal contribution ξ of the variable *a* to the change in the output Ω is shown in purple. The value of ξʹ becomes equal to that of ξ for large values of *K*

This is exemplified by the red line in Fig. 2, which plots the values of Ω on the ordinate as a function of *a* on the abscissa, obtained for the specific input set ***b***_I_ (parameterization). Any situation with well-defined conditions, where *a* and ***b*** are known, is referred to as a ‘state’. These may be the healthy and treated plants’ operational conditions, or the hypothetical scenarios in which limitations are lifted. Only a few states are typically of interest, but, let the number of states be any integer *M*, with *m* being any generic state counted in Roman numbers $(m=\text{I},\text{ II},\text{ III}\dots . M)$.

Any transition between two adjacent states is divided in a number of intervals *K*, being the total number of intervals, with *k* being any generic intermediate interval counted in integers $(k=0, 1, 2, 3\ldots, K)$. For instance, in Fig. 2, the transition between state I and state II is divided in *K* = 4 intervals.

Following Buckley and Diaz-Espejo (2015) the value of a generic input in the interval *k* scales linearly between the values in the two adjacent known states *m* and *m* + I as:

2 $$ a_{{\text{m,k}}} = a_{{\text{m}}} + \frac{k}{K}\left( {a_{{{\text{m}} + {\text{I}}}} - a_{{\text{m}}} } \right), $$

and the calculation is analogous for all other inputs in the set ***b***.

Within the interval *k* to *k* + 1 the ‘marginal contribution of *a* to the change in Ω’ is defined as:

3 $$ \xi \left( a \right)_{\Omega } = f\left( {a_{{{\text{m}},{\text{ k}} + 1}} ,{\varvec{b}}_{{{\text{m}},{\text{k}}}} } \right) - f\left( {a_{{{\text{m}},{\text{k}}}} ,{\varvec{b}}_{{{\text{m}},{\text{k}}}} } \right), $$

and is visualised in Fig. 2 for the transition from *a*_I, 2_ to *a*_I, 3_. This definition of marginal contribution corresponds to that of Eq. (5b) in Buckley and Diaz-Espejo (2015). Equation 3 can be formulated in the analogous form ${{{\xi }^{\prime}(a)}_{\Omega }}_{\phantom{a}}=f\left({a}_{\text{m},\text{k}+1},{{\varvec{b}}}_{\text{m},\text{k}+1}\right)-f\left({a}_{\text{m},\text{k}},{{\varvec{b}}}_{\text{m},\text{k}+1}\right)$ (shown in purple in Fig. 2). Because $f(a,{\varvec{b}})$ is typically non-linear, ${{\xi }^{\prime}(\Omega )}_{a}$ differs from ${\xi (\Omega )}_{a}$ by an error $\varepsilon$, see Deans et al. (2019). For instance, in Fig. 2, ${{{\xi }^{\prime}(a)}_{\Omega }}$ is 30% higher than ${{\xi (a)}_{\Omega }}$. $\varepsilon$ decreases monotonically with* K*, therefore this issue vanishes when a suitably large value of *K* is chosen; in the examples provided *K* = 1000 following Buckley and Diaz-Espejo (2015).

The key metric of contribution analysis is the ‘total contribution of *a* to the change in Ω’, which, in the transition from state I to state *M*, is the sum of the marginal contributions over all intermediate intervals and states:

4 $$ \Xi \left( a \right)_{\Omega } = \mathop \sum \limits_{m = I}^{M} \mathop \sum \limits_{k = 0}^{K} \xi \left( a \right)_{\Omega } . $$

This definition of total contribution corresponds to that of Eq. 5c in Buckley and Diaz-Espejo (2015).

The ‘relative contribution of *a* to the change in Ω’ between the state I and the state M is:

5 $$ \rho \left( a \right)_{\Omega } = \frac{{\Xi \left( a \right)_{\Omega } }}{{\Omega_{M,K} - \Omega_{I,0} }}. $$

The definition of relative contribution corresponds to Eq. 25 in Jones (1985).

The key metric in limitation analysis is the ‘limitation by *a* to the change in Ω’, which, between the state I and the state M is:

6 $$ L\left( a \right)_{\Omega } = \frac{{\Xi \left( a \right)_{\Omega } }}{{\Omega_{{\text{M,K}}} }}. $$

This definition of limitation corresponds to Eq. 13 in Farquhar and Sharkey (1982), Eq. 13 in Jones (1985), and to Eq. 7 in Buckley and Diaz-Espejo (2015) but expressed as percentage therein.

The key metrics in control analysis are absolute and relative sensitivity. The absolute ‘sensitivity of Ω to *a*’, calculated in a particular state and interval (*m*, *k*), is:

7 $$ S\left( \Omega \right)_{a} = \frac{{\xi \left( a \right)_{\Omega } }}{{a_{{{\text{m, k}} + 1}} - a_{{\text{m, k}}} }}. $$

Sensitivity, a finite-difference estimate of $\frac{\partial f}{\partial a}$, corresponds to Eq. (1) in Jones (1985) and to the partial derivatives in Eq. (1) of Buckley and Diaz-Espejo (2015).

Finally, the relative sensitivity better referred to as ‘elasticity of Ω to *a*’, calculated in a particular state (*m*, *k*), is:

8 $$ \eta \left( {\Omega } \right)_{a} = \frac{{\ln f\left( {a_{{{\text{m}},{\text{k}} + 1}} ,{\varvec{b}}_{{{\mathbf{m}},{\mathbf{k}}}} } \right) - \ln f\left( {a_{{{\text{m}},{\text{k}}}} ,{\varvec{b}}_{{{\mathbf{m}},{\mathbf{k}}}} } \right)}}{{\ln a_{{{\text{m}},{\text{k}} + 1}} - \ln a_{{{\text{m}},{\text{k}}}} }}. $$

Elasticity, a finite-difference estimate of $\frac{\partial Ln(f)}{\partial Ln(a)}$ correspond to the relative limitation (*l*_S_, *l*_M_) in Eq. 7 in Grassi and Magnani (2005), to the partial derivatives in Eq. 3 of Buckley and Diaz-Espejo (2015).

There will be as many analogous formulations of Eqs. 1–8 as there are model inputs. All definitions given above are generally valid, but below I will only explore the case in which the output Ω is net photosynthetic CO_2_ uptake (*A*), and the function *f* is hence a model of photosynthesis.

## Applications

### Diffusional and non-diffusional restrictions

Here I will initially use the empirical model of Prioul and Chartier (1977), because it is simple, suitable for all common restriction analyses, and can be used for C_3_ and C_4_ photosynthesis (Bellasio et al. 2018) as well as intermediate types alike. In the formulation of Bellasio et al. (2016b) assimilation is:

9 $$ A_{ } = \frac{{CE \left( {C - {\Gamma }} \right){ } + { }A_{{{\text{SAT}}}} - \sqrt {\left( {CE\left[ {C - {\Gamma }} \right] + A_{{{\text{SAT}}}} } \right)^{2} - (4 \omega A_{{{\text{SAT}}}} CE \left[ {C - {\Gamma }} \right]} )}}{2\omega }, $$

where, *A*_SAT_ is the CO_2_-saturated rate, *CE* is the initial slope, *C* may be *C*_i_ or *C*_M_, Γ is the *x*-intercept, *ω* is defining curvature (Table 1).

**Table 1:** Acronyms, definitions, variables, and units used

Symbol	Definition	Units
*a*	Any generic input of a mathematical model	
*A, A*_op_	Net assimilation, unspecified or measured under ordinary, operational conditions	μmol CO_2_ m^−2^ s^−1^
*A*_SAT_*, A*_SAT_ʹ	CO_2_ saturated *A*, unspecified of after a generic treatment, respectively	μmol CO_2_m^−2^ s^−1^
***b***	A generic set of inputs of a mathematical model which includes all parameters and variables that are not *a*	
*C*_a_	CO_2_ mole fraction outside the leaf	μmol CO_2_ mol air^−1^
*CE, CE*ʹ	Initial slope of the *A*/*C*_i_ curve, unspecified of after a generic treatment, respectively	mol air m^−2^ s^−1^
*C*_i_*, C*_iop_	CO_2_ mole fraction in the substomatal cavity as calculated by the IRGA, unspecified, or under operational conditions	μmol CO_2_ mol air ^−1^
*C*_M_	CO_2_ mole fraction at the mesophyll carboxylating sites	μmol CO_2_ mol air ^−1^
Contribution	Any change in an output variable which is due to a change only in one input, it can be marginal [small csi, ξ, Eq. 3], total [capital csi, Ξ, Eq. 4], or relative [eta, η, Eq. 5]	μmol m^−2^ s^−1^, the relative is dimensionless
*E*	Leaf level water transpiration rate	mmol H_2_O m^−2^ s^−1^
*g*_M_	Mesophyll conductance to CO_2_ diffusion	mol air m^−2^ s^−1^
*g*_S_	Stomatal conductance, here to CO_2_ diffusion also when unspecified, elsewhere often referred to as *g*_SC_	mol air m^−2^ s^−1^
*g*_Tot_	Total conductance to CO_2_ diffusion, the reciprocal of *g*_Tot_ is the sum of the reciprocal of *g*_S_ plus the reciprocal of *g*_M_	μmol m^−2^ s^−1^
*k, K*	Any generic interval between states and the total number of intervals, respectively	
Limitation	The total contribution divided by the maximum value of the function in the interval, Eq. 6	
*L*_NS_	Non-stomatal limitation to photosynthesis	Dimensionless
*L*_S_	Stomatal limitation to photosynthesis	Dimensionless
*m, M*	Any generic state, and the total number of states, respectively	
Restriction	Any generic hindrance to photosynthetic activity	
Elasticity, η	The marginal contribution divided by the value of the variable, relative to the change in input divided by the value of the input, Eq. 8	
Sensitivity, S	The marginal contribution relative to the change in input, Eq. 7	
State	Any condition in which a photosynthetic model can be defined (by knowing *a* and ***b***)	
*μ*	*A*_SAT_ divided by *CE*	
Ω	Any generic output of a mathematical model, a function of *a* and ***b***	
ω	Curvature of the non-rectangular hyperbola describing the* C*_i_ dependence of *A*	Dimensionless

CO_2_ enters the leaf through gaseous diffusion across stomata and the air passages between cells, dissolves in water, and diffuses in solution through the cell wall, plasmalemma and through the mesophyll to reach the Rubisco or phosphoenolpyruvate carboxylase sites of carboxylation, located in the chloroplasts of C_3_ leaves or in the cytosol of C_4_ leaves. The ease of this process is the total conductance ${g}_{\text{Tot}}=\frac{{g}_{\text{S}} {g}_{\text{M}}}{{g}_{\text{S}} + {g}_{\text{M}}}$, where mesophyll conductance (*g*_M_) compounds all processes that are not stomatal. The mesophyll supply function can be written as:

10 $$ C_{{\text{M}}} = C_{{\text{a}}} - \frac{A}{{{ }\frac{{g_{{{\text{S}} }} g_{{\text{M}}} }}{{g_{{{\text{S}} }} + g_{{\text{M}}} }}}} $$

Combining Eqs. (9) and (10) results in a quadratic for *A*:

11 $$ A_{ } = \frac{{CE\left( {\frac{{A_{{{\text{SAT}}}}^{2} }}{{{ }\frac{{g_{{\text{S}}} + g_{{\text{M}}} }}{{g_{{\text{S}}} g_{{\text{M}}} }}}} + C_{{\text{a}}} - {\Gamma }} \right) - \sqrt {\left[ {CE\left( {{\Gamma } - C_{{\text{a}}} - \frac{{A_{{{\text{SAT}}}}^{2} }}{{\frac{{g_{{\text{S}}} + g_{{\text{M}}} }}{{g_{{\text{S}}} g_{{\text{M}}} }}}}} \right)} \right]^{2} - 4 \left( {\omega + \frac{CE}{{{ }\frac{{g_{{\text{S}}} + g_{{\text{M}}} }}{{g_{{\text{S}}} g_{{\text{M}}} }}}}} \right) A_{{{\text{SAT}}}} CE \left( {C_{{\text{a}}} - {\Gamma }} \right)} }}{{2\left( {\omega + \frac{CE}{{{ }\frac{{g_{{\text{S}}} + g_{{\text{M}}} }}{{g_{{\text{S}}} g_{{\text{M}}} }}}}} \right)}}. $$

Equation (11) will be used to evaluate diffusional and non-diffusional restrictions.

### Stomatal limitation

**Fig. 3:**
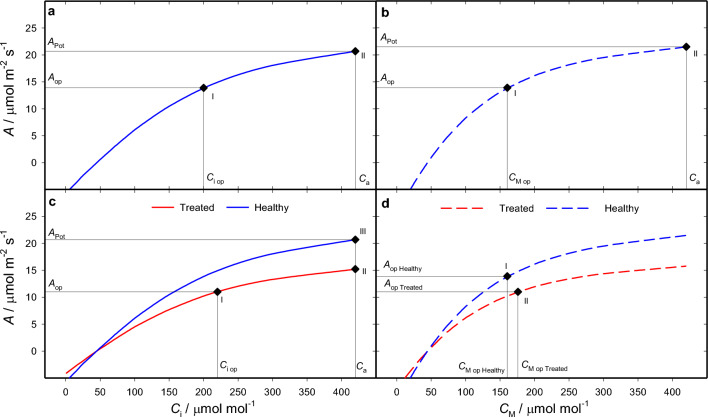
Quantifying limitations and contributions to changes in photosynthesis. *Stomatal limitation*. **a** Shows a photosynthetic model describing the dependence of assimilation (*A*) upon CO_2_ mole fraction in the substomatal cavity (*C*_i_, solid line, Eq. 9) of healthy plants (blue). In this example with hypothetical data, operational conditions correspond to an ambient CO_2_ mole fraction (*C*_a_) of 420 μmol mol^−1^ and a fixed light intensity (the level is irrelevant here). It attains a rate of assimilation (*A*_op_) of 13.9 μmol m^−2^ s^−1^, and has a CO_2_ mole fraction in the substomatal cavity (*C*_i op_) of 200 μmol mol^−1^, corresponding to an operational stomatal conductance to CO_2_ $\left[{g}_{\text{SC op}}=\frac{{A}_{\text{op}}}{{C}_{\text{a}}-{C}_{\text{i op}}}\right]$ of 0.063 mol m^−2^ s^−1^ (Table 2). In the transition between operational State I and hypothetical State II assimilation is driven to its potential as if intercellular spaces were directly exposed to external CO_2_ mole fraction, *g*_S_ becomes high, until *C*_i_ reaches *C*_a_. Assimilation increased from 13.9 to 20.7 μmol m^−2^ s^−1^; the total contribution of *C*_i_ to the change in assimilation $\left[{\Xi ({C}_{\text{i}})}_{A}\right]$ was 6.9 μmol m^−2^ s^−1^; the relative contribution of *C*_i_ to the change in assimilation $\left[{\rho ({\text{C}}_{\text{i}})}_{A}\right]$ was 1 because *C*_i_ was the only input to vary between the two states; the limitation imposed by *C*_i_ to assimilation $\left[{L({C}_{\text{i}})}_{A}\right]$ corresponds to stomatal limitation, was 0.33. ${{S(\Omega )}_{a}}$, and ${{\eta (\Omega )}_{a}}$ are shown in Sheet 1 of Workbook III, calculated for each step of the transition. *Diffusional limitation*. Panel **b** shows the modelled dependence of assimilation (*A*) upon CO_2_ mole fraction in the mesophyll (*C*_M_, dashed line, Eq. 9) of healthy plants. Diffusional limitation is quantified by evaluating transitions from the operational conditions of state I with physiological *g*_M_ and *g*_S_, to a state II where the hindrance to CO_2_ diffusion caused both by the stomata and by the mesophyll is removed by increasing *g*_M_ and *g*_S_ to high values. Assimilation increased from 13.9 in state I, to 21.5 μmol m^−2^ s^−1^ in state II; diffusional limitation was 0.36; details are in Sheet 2 of workbook III. *Stomatal and non-stomatal limitation.* **c** Shows the modelled *A*/*C*_i_ curves of healthy (blue) and treated (red) plants. State I is the operational conditions of the treated plant (diamond ‘I’). State II is where, with otherwise invariant parameterisation, the stomatal barrier is removed by increasing *g*_S_ so that *C*_i_ gM and the value of *C*_a_ of 420 μmol mol^−1^. State III is where* C*_a_ is invariant, while the parameters *A*_SAT_ and *CE* are driven to those of the reference plant. The treated plant had an *A*_op_ of 11 μmol m^−2^ s^−1^, and a *C*_i op_ of 220 μmol mol^−1^, the healthy plant is the same (Table 2). In the transitions, assimilation increased from *A*_op_ of 11 in state I, to *A*_Ca_ of 15.2 μmol m^−2^ s^−1^in state II to *A*_Pot_ of 20.7 μmol m^−2^ s^−1^ in state III. Stomatal limitation, ${{L({\text{g}}_{\text{S}})}_{A}}$, was 0.20; the sum of the other limitations (*A*_SAT_, *CE*, *ω*, and Γ), that is, non-stomatal limitation, was 0.26. Calculations are shown in Sheet 3 of Workbook III. *Contribution analysis.* **d** Shows the modelled *A*/*C*_M_ curves of healthy (blue) and treated plants (red). Healthy plants’* A*/*C*_M_ responses, *A*_op_, *C*_M op_, *g*_SC op_ and *g*_M_ are from previous examples while *g*_SC op_ʹ was 0.055 mol m^−2^ s^−1^. Parameters of the fitted to *A*/*C*_M_ responses are in Table 2. State I and II represent generic operational points of healthy and treated plants, respectively. Stomatal, mesophyll and non-diffusional contribution are quantified in a single transition whereby quantities change linearly between values of healthy and treated plants. The total diffusional contribution was − 0.9 μmol m^−2^ s^−1^, resolved in a stomatal contribution of –0.5 μmol m^−2^ s^−1^, and a mesophyll contribution of − 0.4 μmol m^−2^ s^−1^. The total non-diffusional contribution was –1.7 μmol m^−2^ s^−1^. The calculations are in Sheet 4 of Workbook III.

Stomatal limitation is the reduction of assimilation due to stomatal closure that occurs in a leaf in a real, ordinary operational conditions as compared to a hypothetical case where CO_2_ would freely enter the leaf, thus assuming that the maximum limit of stomatal conductance is infinity. The fixed characteristics of the plant *CE*, Γ, and *ω* (the vector ***b*** in Eq. 1) are found by curve fitting (procedures are available in Workbook I), that is, by inputting the measured value of *C*_i_ in Eq. 9 and adjusting their value until Eq. (9) outputs the value of assimilation that is most similar to that measured. The values fitted to example data are shown in Table 2. Stomatal limitation is lifted in a single transition (number 1 in Fig. 1b) by calculating Eq. (11) in all *K* intervals between state I where *g*_S_ equals *g*_Sop_, and state II, the hypothetical scenario where *g*_S_ is set to an arbitrarily high value (Procedures are available in Workbook II). A numerical example is shown in Fig. 3a. The corresponding calculations are shown in Sheet 1 of Workbook III, where control coefficients (Eqs. 7 and 8) are calculated for each step of the transition. The same procedure can be utilized to compare two known states resulting from any environmental perturbation (transition number 3 in Fig. 1b), as long as they can be described by the same *A/C*_i_ response curve, simply by entering the two relevant *g*_S_ values.

**Table 2:** Synthetic data used in the examples

Measured		Healthy			Treated	
Operational	*A*_op_	*C*_i op_	*C*_M op_	*A*_op_	*C*_i op_	*C*_M op_
	13.9	200	160	11	220	176

*A*/*C* Response	*A*	*C*_i_	*C*_M_	*A*	*C*_i_	*C*_M_
	− 2.59	24	32	− 1.91	24	32
	− 1.22	35	38	− 0.90	35	39
	0.60	50	48	0.44	50	48
	3.00	71	62	2.21	71	62
	6.15	101	84	4.53	101	83
	10.1	145	116	7.42	145	115
	14.4	210	166	10.6	210	164
	17.9	294	243	13.2	294	242
	20.7	420	361	15.2	420	359

Fitted	*C*_i_-based	*C*_M_-based	*C*_i_-based	*C*_M_-based
*A*_SAT_/μmol m^−2^ s^−1^	26	26	19	19
*CE*/mol m^−2^ s^−1^	0.12	0.18	0.09	0.14
*ω*	0.7	0.54	0.7	0.54
Γ/μmol mol^−1^	45	45	45	45

Plants were either untreated or subjected to a hypothetical treatment which reduced the photosynthetic potential. The rates of assimilation (*A*) and CO_2_ mole fraction in the substomatal cavity (*C*_i_) attained in the conditions in which plants were grown are denoted as operational. Gas exchange measurements at the lab bench determined the responses of *A* to *C*_i_. Values of CO_2_ mole fraction at the sites of carboxylation was calculated using mesophyll conductance values of 0.35 and 0.25 mol m^−2^ s^−1^, previously obtained for healthy plants and treated plants, respectively. Assimilation is expressed in μmol m^−2^ s^−1^, CO_2_ mole fraction in μmol mol^−1^. Non-rectangular hyperbolas were fitted either to the *A*/*C*_i_ or to *A*/*C*_M_ curves to obtain two sets of fitted parameters reported at the bottom using the fitting tool of Bellasio et al. (2016b), copied in the sheet ‘AvCi’ of the workbook provided in Supporting Information for convenience

### Diffusional limitation

The analysis of diffusional limitation quantifies the total effect of diffusion from atmosphere to the sites of carboxylation, and requires *g*_M_. This not straightforward to estimate and involves combined measurements of gas exchange and variable fluorescence in C_3_ plants (Bellasio et al. 2016b), or isotopic methods in C_3_ and C_4_ plants (Ubierna et al. 2017; Busch et al. 2020). Diffusional limitation is evaluated in the same way as stomatal limitation, using *C*_M_ in place of *C*_i_. Practically, for each measured value of *C*_i_, the corresponding value of *C*_M_ is calculated as ${C}_{\text{M}}={C}_{\text{i}}-\frac{A}{{g}_{\text{M}}}$. Equation 9 is fitted as described above, this time to the *A*/*C*_M_ response curve obtaining new *C*_M_-based *CE*, and *ω* (Table 2). Equation 11 is calculated in all *K* intervals between state I where *g*_S_ and *g*_M_ take the operational values and, and state II, the hypothetical scenario where *g*_S_ and *g*_M_ are high. Application is exemplified in Fig. 3b and the corresponding calculations are in Sheet 2 of Workbook III.

### Stomatal and non-stomatal limitation

Evaluating the stomatal limitation of treated plants using the procedure shown above (transition number 5 in Fig. 1b) requires knowing the *A*/*C*_i_ responses of the treated plant. When these are impractical or impossible to measure, their parameters need to be estimated. In the method that I recently proposed (Bellasio 2023), the *A*/*C*_i_ curve of the treated plant is obtained by ‘flattening’ the *A*/*C*_i_ curve of the healthy plant (red line in Fig. 2c) to match the pair (*C*_i op_, *A*_op_) measured for the treated plant. The assumptions made are that the curvature* ω* and the CO_2_ compensation point Γ are not affected by the treatment, while the horizontal asymptote *A*_SAT_ and the initial slope *CE* maintain their ratio $\mu ={A}_{\text{SAT}}/CE$ constant. Equation (9) is solved for *CE* as:

12 $$ CE^{\prime}_{ } = \frac{{A \left( {\mu + C - {\Gamma }} \right) + \sqrt {\left[ {A \left( {{\Gamma } - C - \mu } \right)} \right]^{2} - \left[ {4 \omega A^{2} \mu \left( {C - {\Gamma }} \right)} \right]} }}{{2\mu \left( {C - {\Gamma }} \right)}}, $$

*CE*ʹ, the estimated value of *CE* of the treated plant, is directly calculated by inputting measured *A*_op_ and *C*_iop_ or *C*_Mop_ in Eq. (12);* A*_SAT_ʹ, the estimated value of *A*_SAT_ in the treated plant, is, by assumption, *μ* *CE*ʹ. The difference between the potential attained by the treated plant and that of the healthy plant is conventionally referred to as non-stomatal limitation, which can be evaluated in a second transition (number 6 in Fig. 1b). The procedure is shown in Fig. 3c and the corresponding calculations and control analyses are shown for each step in Sheet 3 of workbook III.

An alternative procedure, proposed here anew, lifts limitations in a single transition between the operational conditions of the treated plant and the potential conditions of the healthy plant (number 7 in Fig. 1b).

If *A*/*C*_i_ curves and *g*_M_ are available for both the healthy and the treated plants, diffusional limitation can be resolved in its mesophyll and stomatal components. A first transition may lift diffusional limitation (number 8 in Fig. 1b), while a second transition may lift non-diffusional limitation, as for non-stomatal limitation (number 6 in Fig. 1b). A single transition lifting diffusional and non-diffusional limitation concurrently is also possible (not shown in Fig. 1). Also, these two alternatives are new to this study.

### Stomatal, mesophyll and non-diffusional contributions

If *A*/*C*_i_ curves and *g*_M_ are available for both the healthy and the treated plants, the effect of the treatment can be resolved in its biochemical, mesophyll and stomatal components in a single transition (number 4 in Fig. 1b). This is essentially a simple generalisation of the method of Buckley and Diaz-Espejo (2015). A numerical example is shown in Fig. 3d, and the corresponding calculations and control analysis are in Sheet 4 of Workbook III. If *g*_M_ for the treated plant is not available, the corresponding transition (number 3 in Fig. 1b) can be implemented in with the same procedure, using the *C*_i_-based parameterisation, and by setting an arbitrarily high value for *g*_M_. This variant is new to this study.

### Control analysis

The extent to which assimilation is controlled by a marginal change in the underpinning variables (typically, *g*_S_ and *g*_M_ alongside other inputs in ***b***) is quantified by sensitivity. The strength of this analysis lies in being univocal in any given state, allowing for independent evaluation of plants (e.g. healthy plants under ordinary operational conditions) and a posteriori comparison (e.g. across species in the phylogeny (Gago et al. 2019)).

This analysis can be implemented using the logic of previous examples by imagining that the transition between states becomes arbitrarily small. In practice, Eq. (11) is calculated for the state of interest, and that in which *g*_S_ and *g*_M_ (alongside *A*_SAT_, *CE*, ω, and Γ, or a subset thereof) differ by a marginal increment ($i=\frac{da}{a}$; for *i* → 0, $\frac{da}{a}=dLna$). Calculations for the healthy plant (Table 2) with *i* = 0.0001 applied to *g*_S_, *g*_M_, *A*_SAT_, and *CE* are included in Sheet 7 of Workbook III. The key outputs are absolute and relative sensitivity (in the example, ${S(A)}_{{g}_{\text{S}}}$ averaged 113 μmol mol^−1^, ${\eta (\text{A})}_{{g}_{\text{S}}}$ averaged 0.5), and the relative change in assimilation, which adds to unity when compounding all inputs (in the example, ${\rho (A)}_{{g}_{\text{S}}}$ was 0.5 ${\rho (A)}_{{g}_{\text{M}}}$ was 0.09, ${\rho (A)}_{{A}_{\text{SAT}}}$ was 0.17, and ${\rho (A)}_{CE}$ was 0.25). In small transitions, ${\eta (\text{A})}_{{g}_{\text{S}}}$ and ${\eta (\text{A})}_{{g}_{\text{M}}}$ correspond to *l*_S_ and *l*_M_ calculated after Grassi and Magnani (2005) while *L*(*g*_S_) and *L*(*g*_M_) correspond to *S*_L_ and *MC*_L_ in their Eq. (6).

### Light and non-light restrictions

#### Light limitation

Light limitation analysis can be utilized to quantify the reduction of assimilation due to shading, relative to a hypothetical case where the leaf would be exposed to maximum lighting. Similar to the procedure used for stomatal limitation analysis, light response curves are measured under specific growth conditions (CO_2_ concentration, temperature, etc*.*). Then, the dependence of assimilation on light intensity is described with the model of Prioul and Chartier (1977) after Bellasio et al. (2016b) as:

13 $$ GA_{MOD} = \frac{{Y\left( {CO_{2} } \right)_{{{\text{LL}}}} PPFD + GA_{{{\text{SAT}}}} - \sqrt {\left( {Y\left( {CO_{2} } \right)_{{{\text{LL}}}} { }PPFD + GA_{{{\text{SAT}}}} } \right)^{2} - 4{ }m{ }Y\left( {CO_{2} } \right)_{{{\text{LL}}}} { }PPFD{ }GA_{{{\text{SAT}}}} } }}{{2{ }m}}, $$

Equation 13 (coded in the ‘GAvPPFD’ Sheet of Workbook I) is fitted to the experimental data to derive the parameters for the healthy plant. Light limitation is quantified by evaluating a single transition between operational conditions, and where light limitation is assumed to be absent (number 1 in Fig. 1c), using the procedures described in Workbook II. A worked example is provided in Sheet 6 of Workbook III.

#### Light contribution

The previous procedure is apt for analysing light contributions between two real conditions of interest by specifying the second condition in the parameterization of State II (number 2 in Fig. 1c).

#### Light and non-light limitations

The analysis of light and non-light limitations can be used to distinguish the effect on assimilation directly due to shading from that due to the downregulation of the biochemical potential that occurred in a treated plant. Analogous to the approach used to resolve diffusional and non-diffusional limitations, two variants are possible. In the first case, light limitation is lifted first (number 3 in Fig. 1c), and the remainder is the difference with the potential of the healthy plant (number 4 in Fig. 1c). Alternatively, limitations are lifted in a single transition (number 5 in Fig. 1c, exemplified in Sheet 7 of Workbook III).

## Mechanistic models

Mechanistic models are typically more complicated than empirical, rely on a number of assumptions, are valid only when assumptions hold (e.g. a C_4_ model is not meaningful for mechanistically describing C_3_ plants) and they are less flexible in data fitting. Unless resolving specific biochemical processes is required, the simple empirical models shown above suffice and are therefore recommended, and the provided spreadsheets can be used without modification.

Mechanistic parameters obtained from previous experiments or literature, can be approximated to empirical parameters as follows: *CE* corresponds to $\frac{{V}_{\text{MAX}}}{K}$ (where *V*_MAX_ may be Rubisco or PEP carboxylase maximum rates and *K* is the corresponding Michaelis–Menten constant, adjusted for oxygen concentration in Rubisco’s case); ${A}_{\text{SAT}}\approx \frac{J}{4}-{R}_{\text{L}}$, where *J* is the rate of electron transport, and *R*_L_ is light respiration, or, for C_4_ plants ${A}_{\text{SAT}}\approx \frac{{J}_{\text{ATP}}}{5.5}-{R}_{\text{L}}$, where *J*_ATP_ is the rate of ATP production; Γ is typically available in the literature (alternatively, it can be calculated from *R*_L_ and *K* using, for instance, Eqs. 2.38 or 4.50 in von Caemmerer (2000) for C_3_ plants or C_4_ plants, respectively); whilst the curvature ω can typically be assumed as 0.7. For a more precise conversion, synthetic *A*/*C* response curves (10–15 dummy values of *C*_i_—or *C*_M_—are inputted the same mechanistic model formulations from which the mechanistic parameters were originally derived, parameterised with those original parameters, to output 10–15 values of *A*; these synthetic *A*/*C* curves are fitted to derive empirical parameters), can be refitted and then used to evaluate restrictions, as described above. Alternatively, mechanistic models can be directly used in place of Eq. 11, but in this case the spreadsheets need to be modified. Well-thought-out procedures are needed, designed specifically to address the experimental question.

Mechanistic models may be used when resolving non-diffusional processes is of interest. For instance, they can distinguish between restrictions due to light reactions or carbon metabolism. The method presented by Buckley and Diaz-Espejo (2015) differs from the analysis of mesophyll, stomatal, and non-diffusional contributions exemplified above only in that it uses an update of the C_3_ model by Farquhar et al. (1980). This model has previously been used for the analysis of limitations imposed by Rubisco activation in its enzyme limited submodel (Deans et al. 2019) or in the full formulation (Taylor and Long 2017), and can also be used to quantify limitations imposed by electron transport and triose phosphate utilisation (Buckley and Diaz-Espejo 2015). However, this method is meaningful only for C_3_ plants; obtaining specific values for the Rubisco Michaelis–Menten constants for carboxylation and oxygenation (*K*_C_ and *K*_O_) for measured leaves may not be readily achievable, and the cut-off point for enzyme and light limitation must be assumed.

A C_4_ mechanistic model may serve for the quantification of restrictions due to diffusion through stomata or the mesophyll, as well as restrictions caused by other factors like light intensity, the light-saturated rate of ATP generation (*J*_SAT_), the curvature of the light dependence of ATP generation (θ), the initial quantum yield for ATP generation *Y*(*J*_ATP_)_LL_, respiration in the light, Rubisco and phosphoenolpyruvate carboxylase CO_2_ saturated rate of carboxylation (*V*_CMAX_ and *V*_PMAX_), the factor that partitions ATP between PEP regeneration and the remainder of photosynthetic activity (*x*), and bundle sheath conductance (*g*_BS_). For example, it can be used to determine the contribution of light versus that of *g*_BS_ in the reduction of assimilation for plants grown under shading (Bellasio and Griffiths 2014b). For a corrected formulation refer to Bellasio et al. (2017).

For simulating more complex scenarios and addressing a broad range of questions relating carbon and light reactions I recommend utilizing the Bellasio and Farquhar (2019) and Bellasio and Ermakova (2022) analytical models. The analytical model of Bellasio and Farquhar (2019) allows the quantification of limitations imposed by a wide range of real or imagined scenarios, in any photosynthetic type (C_3_, C_2_, C_2_ + C_4_, C_4_), intermediates thereof, and engineered transitions between them, stomatal opening and closure, different stomatal responsiveness to biochemical forcing or drought, and a wide range of characteristics of the electron transport chain (*e.g.* shifting the ratio between ATP and NADPH production by modifying the fraction of cyclic electron flow or the NDH pathway, etc.). The analytical model of C_4_ photosynthesis of Bellasio and Ermakova (2022) relates to leaf anatomy following Bellasio and Lundgren (2016) and explicitly accounts for light harvesting partitioning between cell compartments. It was specifically developed for studying differences between C_4_ photosynthetic subtypes, and maintains validity at very low irradiance. It features two electron transport chains, C_4_ cycle reactions, and C_3_ cycle reactions accommodating a variety of possibilities for parameterisation.

## Discussion

I set out to generalise the analyses of photosynthetic restrictions, providing a set of definitions that integrate all common and several new procedures, which I illustrated using synthetic data.

The new framework (Eqs. 2–8, and Fig. 1) is valid for all photosynthetic types and models, and it comprehensively quantifies limitations, contributions, and photosynthetic control (sensitivity and elasticity) within a single procedure. This represents a significant advancement since the most widely used protocols—control analysis (Grassi and Magnani 2005), contribution analysis (Buckley and Diaz-Espejo 2015) and limitation analysis (Farquhar and Sharkey 1982)—were traditionally independent. This method is formulated using the principles of Buckley and Diaz-Espejo (2015), and I demonstrated its convergence with Farquhar and Sharkey (1982), and for small transitions with Grassi and Magnani (2005) (see ‘Control analysis’ section). For complete generalisation, I also formulated within the new framework the method of Björkman et al. (1980), which, although widely used, had never been formalised (Fig. S1a, and discussed below).

The new framework avoids two erroneous practices: that of approximating curves with lines in finite intervals (see Introduction), and that of overestimating their slopes. The latter practice was introduced by Tomás et al. (2013) and gained traction in later studies, for instance by Tosens et al. (2016); Carriquí et al. (2015); Carriquí et al. (2019); Perera-Castro et al. (2022); Hu et al. (2024). The slope of the *A*/*C*_M_ response curves required for the linear approximation of the infinitesimal analysis of Grassi and Magnani (2005) was derived under low CO_2_ concentrations—where it is typically several-fold higher than under ordinary operational conditions. This inevitably leads to largely overestimating the importance of stomata and mesophyll diffusion in restricting assimilation—and has likely brought to overstating their importance.

When only one quantity varies, procedures are unambiguous. Many of the applications I presented, such as diffusional limitation and light limitation analyses, are evaluated by formulating calculations so that only one quantity (variable *a* in Eq. 1) varies between transitions, while the other inputs (vector ***b*** in Eq. 1) remain constant. In all those cases the method is unequivocal, and equivalent both for limitation and contribution analysis.

When multiple quantities vary, methodological variants are possible (the number of variables changing raised to the power of the number of transitions), but it is still generally straightforward to define how to proceed, or results will not differ between alternatives, as I will now discuss.

In classical contribution analysis, it had been proposed to assume that stomata respond before any change in leaf biochemical processes occurs, or the reverse. Methodological papers seem to prioritise cases where the differences between alternative approaches were large; for instance Assmann (1988) reported a fivefold difference. However, in common applications, the differences may not be as pronounced. With the data of the examples, the discrepancy between path-dependent alternatives (shown in Figure S1b and calculated in sheets 9 and 10 of Workbook III) was relatively small, about ± 20%, of the independent path. Nevertheless, path dependent methods are not recommended. While their assumptions may partially hold under specific conditions, such as when stomatal adjustment is much slower than photosynthetic activation (Deans et al. 2019), they are generally invalid. In fact, during the imposition of a treatment, photosynthetic characteristics typically respond progressively, driven by a common factor—the treatment—and are therefore not independent. Though, since all changes occur concurrently, as proposed by Buckley and Diaz-Espejo (2015) all quantities can be presumed to change linearly between their initial and final values (number 3 in Fig. 1b), thus eliminating any ambiguity in the path to follow.

In the analysis of limitations in treated plants, there is the possibility to opt for a single or two-tiered transition. Typically, the experiment and its research questions will naturally determine the appropriate method to follow. Plants are often subject to permanent or mid-term acclimation treatments (e.g., differing nitrogen levels, watering levels, growth light conditions, growth CO_2_ levels, soil types, fertilisation, mycorrhization, etc.), or differ by any genetic trait, whether natural or engineered. These traits develop over time and are not subject to short-term changes, and it is possible to devise a hypothetical scenario whereby limitations are lifted instantaneously and independently of the treatment, assuming the characteristics of the plants remain constant. Applying this principle involves evaluating two transitions (numbers 5 and 6 in Fig. 1b), which is the conventional approach used in the literature. I presented a novel single-transition variant (number 7 in Fig. 1b), which has the benefit of being univocally defined. Strikingly, the distinction between single or two-tiered transition is merely conceptual, as the results do not differ. Under current assumptions—that inputs scale linearly (Eq. 2) and diffusional limitations are fully lifted in the idealised state—the single-transition variant accurately tracks the two-transition variant. (This can be verified by typing 1 in cell C1 and “n” in cell F10 in Sheet 3 of Workbook III). Therefore, unless additional assumptions are introduced, variants can be used interchangeably.

Sometimes it may be difficult to determine the characteristics ***b*** of the treated plants. Through modern gas exchange equipment (Stinziano et al. 2017) and elegant routines, Taylor and Long (2017) measured rapid* A*/*C*_i_ responses in real time during the treatment’s course. However, often this is impractical; in which case the characteristics of treated plants must be estimated. A simple and widely used method is that of Björkman et al. (1980), in which the estimation follows the assumption that lifting stomatal limitation in the treated plant would result in the same increase in assimilation as in the healthy plant (details and mathematical formalisation in Fig. S1a). This simplification is generally acceptable for C_4_ plants because their *A/C*_i_ curves are flat for *C*_i_ > *C*_iop_. For C_3_ plants, it may be acceptable only when the treatment effect is small, but stomatal limitation is overestimated when the assimilatory potential is severely reduced by the treatment. The method I followed here to derive an analytical solution is based on Bellasio (2023). It involves flattening the *A/C*_i_ curves of treated plants, under the assumption that *CE* and *A*_SAT_ maintain their ratio (Eq. 12). This corresponds mechanistically to fixing the *J*/*V*_MAX_ ratio, and may not have general validity, as the treatment’s effect on parameters may be species- and treatment-dependent. The coded procedure allows to input the actual ratio, which should be preferred if available. Γ and ω are maintained invariant based on the rationale that they are underpinned by enzymatic properties (see ‘Mechanistic models’ above), and indeed measurements show that, for instance, they did not significantly change in both C_3_ and C_4_ plants during seasonal drought (Bellasio et al. 2022).

The analysis of light and non-light restrictions is original to this study. Lifting light limitation may not involve exposing leaves to infinite light intensity but just to full sunlight, making it difficult to pinpoint the real versus hypothetical conditions, creating confusion between contribution and limitation analyses. The experimental question and the timing of acclimation may once again guide the choice. A decrease in assimilation may result from a combination of direct and indirect effects of shading, such has the decrease in biochemical potential that occurs in a leaf shaded by the overgrowth of a new canopy (Bellasio and Griffiths 2014a). Because these responses are not independent and occur over time, in this case the analysis of contributions in a single transition may be appropriate. Alternatively, if the treatment has affected the biochemical potential independently of light, such as in cases of pathogen attack or nitrogen level changes (Dewar et al. 2012), then limitation can be lifted independently and instantaneously therefore an analysis with a two-tiered transition may be preferable.

## Conclusion

I urge the community to critically reconsider the relative importance of stomatal, mesophyll, and non-diffusional restrictions, especially when they were evaluated using the linear approximation in finite intervals by Grassi and Magnani (2005), and the initial slopes of the *A*/*C*_M_ curves by Tomás et al. (2013), which are erroneous.

## Supplementary Information

Below is the link to the electronic supplementary material.

Supplementary file1 (XLSX 2050 KB)

Supplementary file2 (XLSX 2056 KB)

Supplementary file3 (XLSX 12186 KB)

Supplementary file3 (DOCX 29 KB)

## Data Availability

The workbooks coding curve fitting, procedures for restriction analysis and worked examples are available in the Supporting Information of this article. A curated version is available on GitHub at the link [https://github.com/chandrabellasio/Quantifying-Photosynthetic-Restrictions-]. The C_3_ and C_4_ models of Farquhar et al. (1980) and Berry and Farquhar (1978) in the formulations of Bellasio et al. (2017) can be made available upon request. Spreadsheets coding the Bellasio and Farquhar (2019) and Bellasio and Ermakova (2022) analytical models are available in the Supporting Information of the original publications. No datasets were generated or analysed during the current study.
