# Supplementary material for: Quantifying photosynthetic restrictions
Source: Photosynth Res. 2025 Feb 18;163(2):19. doi: 10.1007/s11120-024-01129-y (PMC11835928; doi:10.1007/s11120-024-01129-y)
Supplement: Supplementary file 4 — Supplementary file3 (DOCX 29 KB) [file 11120_2024_1129_MOESM4_ESM.docx]

**Figure S1. Alternative path dependent methods**Panel **A** shows the method of [Björkman et al. (1980)](#_ENREF_15). The *A*/*C*_i_ curve for the treated plant (in red) is approximated with that of the healthy plant (in blue) for *C*_i_ >*C*_i op_, translated vertically to match the operational assimilation of the measured plant as:

| $f\left( C,\boldsymbol{b}\boldsymbol{'} \right)\approx f\left( C,\boldsymbol{b}^{\boldsymbol{''}} \right)-\left[ f\left( C_{i op}, \boldsymbol{b}\boldsymbol{''} \right)-f\left( C_{i op},\boldsymbol{b}^{\boldsymbol{'}} \right) \right]$, | S1 |
| --- | --- |

where ***b***′ and ***b***′′ are the set of parameters of the treated, and healthy plant, respectively; $f\left( C, \boldsymbol{b}\boldsymbol{'} \right)$ is the *A*/*C* function to be estimated; $f\left( C,\boldsymbol{b}\boldsymbol{''} \right)$ is the known *A*/*C* function with the parameters ***b***′′ determined for the healthy plant; the expression in square brackets is the down-translation (Figure 4A). $A_{\mathrm{op}}$ is the rate of assimilation of the treated plant in operational conditions,$f\left( C_{\mathrm{iop}},\boldsymbol{b}^{\boldsymbol{'}} \right)$ in Eqn S1; $A_{Pot Ci}$, is the rate of assimilation that the healthy plant would have if *C*_i_ were that of the treated plant in the operational condition *C*_iop,_, $f\left( C_{\mathrm{iop}}, \boldsymbol{b}\boldsymbol{''} \right)$ in Eqn S1; $A_{\mathrm{Pot}}$is that a healthy plant would have in absence of stomatal barrier, $f\left( C_{a}, \boldsymbol{b}\boldsymbol{''} \right)$ in Eqn S1. Limitations are evaluated by removing stomatal limitation first in the transition between state I and state II, and then by removing non-stomatal limitation in the transition between state II and state III. The conventional three point calculation ([Bellasio et al. 2023](#_ENREF_13)) gives the same results as the current framework: the total stomatal contribution ${{\Xi(C_{i})}_{A}}$ is $A_{\mathrm{Pot}}- A_{Pot Ci}$, and the total non-stomatal contribution ${\Xi(L_{\mathrm{NS}})}_{A}$ is $A_{Pot Ci}- A_{\mathrm{op}}$. Calculation of Eqn S1 for each step of the first transition is shown in Sheet 8 of Workbook III.

Panel **B** shows two path-dependent variants of contribution analysis where a healthy plant (in blue) undergoes a generic treatment that restrict assimilation (in red). State I and III represents a generic operational point of the treated plant, and healthy plant, respectively, while state II is an intermediate state through which the transition is assumed to occur. The first path assumes that in response to the treatment stomata respond before any change in leaf biochemical processes occurs, that is, a transition from state III to state II, with reduced *g*_S_, and then to state I, with lower photosynthetic potential but equal *g*_S_. The second variant assumes that biochemical regulation occurs before stomata respond, that is, a path through state IIʹ where *g*_S_ equals that of the healthy plant, while the parameterization corresponds to that of the treated plant. In the path ‘stomata first’ during the transition between state I and state II, ${\Xi(g_{S})}_{A}$ was -1.1 μmol m^-2^ s^-1^. In the path ‘mesophyll first’ during the transition between state II and state III, ${\Xi(g_{S})}_{A}$ was -0.7 μmol m^-2^ s^-1^. The calculations are in Sheets 9 and 10 of Workbook III.
